# Correction: Use of biomaterials for sustained delivery for anti-VEGF to treat retinal diseases

**DOI:** 10.1038/s41433-020-0980-3

**Published:** 2020-05-22

**Authors:** Ivan Seah, Xinxin Zhao, Qianyu Lin, Zengping Liu, Steven Zheng Zhe Su, Yew Sen Yuen, Walter Hunziker, Gopal Lingam, Xian Jun Loh, Xinyi Su

**Affiliations:** 1 Department of Ophthalmology, National University Hospital Singapore, Singapore, Singapore; 2 Institute of Molecular and Cell Biology (IMCB), Agency for Science, Technology and Research (A*STAR), Singapore, Singapore; 3 Institute of Materials Research and Engineering (IMRE), Agency for Science, Technology and Research (A*STAR), Singapore, Singapore; 4 Department of Ophthalmology, Yong Loo Lin School of Medicine, National University Singapore, Singapore, Singapore; 5 Singapore Eye Research Institute, Singapore, Singapore; 6 Lee Kong Chian School of Medicine, Nanyang Technological University, Singapore, Singapore; 7 Department of Physiology, National University of Singapore, Singapore, Singapore

**Keywords:** Implants, Macular degeneration, Retinal diseases, Antibody therapy, Drug therapy

Correction to: *Eye*

10.1038/s41433-020-0770-y

The original article was published with errors pertaining to the details of the Port Delivery System (PDS) clinical trials.

In paragraph 3 of the section titled “Non-Biodegradable Implants”, several statements require correction. These statements are detailed below along with the corresponding corrections.

1. “The PDS had a mean refill time of 15 months”.
  In the Phase II LADDER trial, the PDS had a median refill time of 15 months.

2. “The trial is scheduled for completion in May 2022”.
  The estimated completion date of the Phase III ARCHWAY trial (ClinicalTrials.gov NCT03677934) should be April 2022.
3. “Concurrently, a long-term efficacy and safety trial, the PORTAL trial (ClinicalTrial.gov ID: NCT03683251), will also evaluate 500 participants for 144 weeks with 24 weeks of periodic refills”.
  Participants in the Phase III PORTAL extension trial will receive refills every 24 weeks for up to 144 weeks.
4. The following statement was made “This trial is scheduled for completion in June 2022.” —The estimated completion date of the Phase III PORTAL trial (ClinicalTrials.gov NCT03683251) should be January 2022.

All of these errors have been corrected in the HTML and PDF versions of this article.

